# China industrial environmental database 1998–2015

**DOI:** 10.1038/s41597-022-01362-x

**Published:** 2022-06-01

**Authors:** Haoqi Qian, Feizhou Ren, Yanran Gong, Rong Ma, Wendong Wei, Libo Wu

**Affiliations:** 1 Institute for Global Public Policy and MOE Laboratory for National Development and Intelligent Governance, Fudan University, Shanghai, 200433 China; 2 LSE-Fudan Research Centre for Global Public Policy, Fudan University, Shanghai, 200433 China; 3 Shanghai Pudong Development Bank, Shanghai, 200002 China; 4 School of Economics, Fudan University, Shanghai, 200433 China; 5 School of Data Science, Fudan University, Shanghai, 200433 China; 6 School of International and Public Affairs, Shanghai Jiao Tong University, Shanghai, 200030 China; 7 SJTU-UNIDO Joint Institute of Inclusive and Sustainable Industrial Development, Shanghai Jiao Tong University, Shanghai, 200030 China; 8 Institute for Big Data, Fudan University, Shanghai, 200433 China; 9 Shanghai Institute for Energy and Carbon Neutrality Strategy, Fudan University, Fudan University, Shanghai, 200433 China

**Keywords:** Environmental economics, Environmental impact, Economics, Interdisciplinary studies, Sustainability

## Abstract

There has been a rapid-growing trend in studying China’s environmental problems in the past decade. However, the existing environmental statistics data are far from meeting researchers’ requirements. The biggest problem is that the official environmental statistics data are only provided at either regional level or sectoral level. Considering the huge heterogeneities in different regions and sectors, researchers are unable to conduct comprehensive policy evaluations. In this study, we constructed the time-series industrial environmental database for China (CIED) at both regional and sectoral level. The database includes totally 31 regions and four types of pollutants: chemical oxygen demand (COD), sulphur dioxide (SO_2_), ammonia-nitrogen (NH_3_-N), and nitrogen oxide (NO_X_). This study also clarifies several important concepts for researchers to better understand China’s official environmental statistics data.

## Abstract (summary)

**Table:** 

Measurement(s)	environmental pollution
Technology Type(s)	computational modeling technique
Sample Characteristic - Location	China

## Background & Summary

China’s environmental problem is one of the most important issues accompanying with its rapid industrialization. Although the situation has been greatly improved in the past decade, the whole nation, especially in certain regions, is still facing severe environmental problems such as air pollution issue, water pollution and so on. Since 2000s, there is a growing number of evidence-based literature focusing on China’s environmental problems. Topics of these literature include discussions of China’s environmental Kuznets curve^1–5^, analyses of environmental protection performance^6–9^ and evaluations of environmental policies^10,11^. Most of these studies use either regional-level or sector-level environmental data that are retrieved from official statistics to conduct analyses, which fail to take both regional and sectoral heterogeneities into consideration simultaneously. The main reason is that the official statistics such as China Statistical Yearbook on Environment only report provincial aggregated pollution data and sectoral aggregated pollution data. Attempts to partly overcome this problem are to use micro-level data such as firm-level or plant-level data, and cases can be seen in several recent studies^12–15^. These studies provide insights into firms’ polluting behaviours and also do provide new evidence in understanding regional and sectoral variations in China.

There are two main drawbacks in using micro-level data, especially firm-level data, to study the environmental issues. Firstly, a certain number of observations will be dropped when constructing the balanced panel dataset which plays an important role in most empirical studies. Sample representativeness will be further weakened if researchers match different firm-level datasets in order to obtain more variables. As a result, other important topics such as structure effect, entry effect and exit effect can not be investigated at the macro-level. Secondly, many important influence factors are only available and applicable in regional level or sectoral level, and micro-level data only contains individual firm specific characteristics. But most of current research interests and policy needs are at the macro-level. In this case, firm-level analysis may unable to deliver desired results and policy implications at the macro-level. Due to the above two reasons, estimating environmental data at both regional level and sectoral level will undoubtedly contribute to the existing empirical works. For example, the two-dimensional data have great potentials to boost environmental policy evaluations by using treatment effect analyses such as difference-in-difference (DID) model^16–18^ and synthetic control method (SCM)^4,19,20^, which have gained much popularity in this field in the past decade. The second direction of the application is conducting environmental efficiency analyses such as data envelope analysis (DEA) and stochastic frontier analysis (SFA), by viewing pollutions as undesirable products^21–24^. Furthermore, two-dimensional environmental data can also be used to construct environmentally extended multi-regional input-output (EE-MRIO) tables^25–27^, as well as provide more accurate estimates for parameters^28^, which are extremely useful in conducting complex economic system analyses such as computable general equilibrium (CGE) modelling works.

In the China Industrial Environmental Database (CIED), there are 31 regions (excluding Hongkong, Macao, and Taiwan) and 39 industrial sectors. For each sector in each region, we provide environmental data of four types of pollutants: chemical oxygen demand (COD), sulfur dioxide (SO_2_), ammonia-nitrogen (NH_3_-N), and nitrogen oxide (NO_X_). The database contains discharge and removal data for all four pollutants. Due to the data availability, data for COD and SO_2_ are available from 1998 to 2015, data for NH_3_-N is available from 2001 to 2015 and data for NO_X_ is available from 2006 to 2015. The time period covers last three years of the ninth Five-Year Plan and the successive three complete Five-Year Plan periods. Since Five-Year Plans play great roles in the policy-making procedure, the database can be used to analyze a wide range of institutional and policy adjustment issues during this period.

## Methods

In this study, we combine the bottom-up method and top-down method to construct the two-dimensional environmental database. In the first step, the bottom-up method uses firm-level environmental data calculate pollutants’ discharge and removal amount by region and by sector. In the second step, the top-down method collects total amount of pollutants discharged and removed for each region or for each sector. In the final step, the cross-entropy method is used to balance the two-dimensional matrix in each year.

### Aggregation of micro-level environmental data

Micro-level environmental data are retrieved from China’s Environmental Statistics Database (CESD). The CESD is a micro-level database compiled by China’s Ministry of Environmental Protection (MEP) which is used as the data basis for publications of China’s official environmental statistics such as China Statistical Yearbook on Environment, China Environment Yearbook and Annual Statistic Report on Environment in China. This study uses the industrial firm-level data from the CESD and we call it China’s Industrial Environmental Statistics Database (CIESD). The CIESD is a newly released database available to researchers which covers all major industrial emission sources in China. This database has already been used and explained in details in several recent studies^14,15,29,30^.

Table 1 reports the number of firms covered in our database by region and year. We should pay some special attentions when using the database. Totally 20 cells in Table 1 are zero or close to zero for all regions, which means these observations are completely or almost missing. The overall data coverage rates in 1998 and 1999 are 75.38% and 91.97% respectively, which are not so perfect but acceptable for this study. In 2006, there are totally 1784 firms which cannot be categorized to any region by using 6-digit administrative division code. After carefully checking these observations manually, we find they are all duplicated observations for Anhui province. After comparing data of these duplicated observations, we just keep one unique observation with useful information. For the period during 2006 and 2010, environmental statistical data for thermal power plants are collected as a separate database, which is not included in our database. Total number of thermal power plants are also reported in Table 1. Therefore, if we subtract numbers of thermal power plants in the period during 2006 and 2010, the overall data coverage rates from 2000 to 2014 are quite close to 100% which means a quite good sample representativeness.

**Table 1:** Distribution of CIESD firms across 31 regions, 1998–2014.

	1998	1999	2000	2001	2002	2003	2004	2005	2006	2007	2008	2009	2010	2011	2012	2013	2014
Beijing	0	1,015	1,517	1,133	1,004	4	838	794	730	850	787	808	711	914	896	932	1,534
Tianjin	0	0	1,228	1,839	1,596	1,617	1,615	1,372	1,765	1,610	1,767	1,871	1,940	1,988	2,021	2,657	3,315
Hebei	2,818	2,563	2,671	3,011	2,724	2,634	2,865	2,844	2,871	4,958	5,549	5,677	5,802	10,524	10,074	10,488	10,856
Shanxi	2,891	2,769	2,759	3,395	3,482	3,375	3,189	3,116	3,304	3,817	3,918	3,661	3,942	6,464	6,367	6,258	6,509
Inner Mongolia	1,163	1,134	1,022	927	903	962	1,192	1,266	1,282	1,823	2,211	2,137	2,259	3,187	3,025	3,110	3,724
Liaoning	6	2,590	2,508	2,810	2,595	2,597	2,495	2,988	3,976	4,768	4,912	4,885	4,559	6,586	6,316	6,305	8,366
Jilin	1,062	1,058	1,066	989	897	881	844	808	749	845	941	963	967	1,489	1,466	1,483	1,540
Heilongjiang	108	1,839	1,770	1,544	1,596	1,619	1,506	1,414	1,314	1,417	1,500	1,537	1,480	2,083	1,927	1,884	1,914
Shanghai	2,215	2,052	2,000	1,938	1,808	1,732	1,597	1,575	1,763	1,721	1,802	1,749	1,850	2,283	2,158	2,089	2,228
Jiangsu	5,557	6,235	6,369	4,898	5,068	5,604	5,518	5,800	6,081	7,674	7,896	7,905	7,887	11,291	11,107	10,743	10,731
Zhejiang	4,660	4,751	5,433	5,537	5,634	5,664	5,749	5,848	6,199	9,999	10,119	10,889	10,767	13,931	13,541	13,211	12,342
Anhui	1,978	1,776	1,981	1,728	1,691	1,714	1,644	1,586	1,788	2,717	2,944	3,150	4,112	8,552	8,403	8,365	8,402
Fujian	3,503	3,694	3,737	3,213	3,043	3,184	3,098	3,135	3,108	6,196	6,153	6,091	6,053	5,802	5,740	5,755	5,767
Jiangxi	0	0	1,107	942	992	1,042	1,116	1,160	1,285	2,453	2,861	2,970	3,308	5,197	5,118	5,115	5,682
Shandong	1,754	5,446	5,425	5,246	5,107	5,186	4,994	5,038	5,014	5,838	5,569	6,330	6,052	8,008	7,791	7,708	7,911
Henan	2,510	3,416	3,611	4,108	2,866	3,158	4,047	3,402	3,737	4,644	4,101	4,280	4,333	6,678	6,550	6,494	6,967
Hubei	2,302	2,046	2,187	2,222	2,236	2,589	2,283	2,312	2,194	2,388	2,505	2,518	2,613	3,911	3,699	3,590	3,912
Hunan	3,526	3,021	2,845	2,839	2,823	3,156	3,139	3,015	2,716	3,310	3,670	3,852	3,906	5,008	4,800	4,668	4,831
Guangdong	5,507	5,451	6,500	7,037	7,068	7,017	6,724	6,376	6,702	12,948	12,974	11,785	11,939	15,907	14,890	14,959	15,145
Guangxi	1,716	1,769	1,885	1,886	1,687	1,734	1,719	1,730	1,785	4,333	4,795	4,631	4,430	3,565	3,517	3,532	3,464
Hainan	287	298	282	294	293	283	295	282	242	282	338	357	327	483	460	458	496
Chongqing	1,241	1,336	1,240	1,449	1,432	1,476	1,367	1,388	1,916	2,453	2,758	2,875	2,458	3,212	3,107	3,145	3,677
Sichuan	3,268	3,458	3,463	3,465	3,638	3,965	4,020	4,136	5,370	6,217	5,904	5,516	6,571	7,720	7,548	7,510	7,750
Guizhou	2,273	2,021	2,148	2,567	2,636	2,590	2,949	2,990	2,832	2,942	3,504	3,375	3,450	4,131	3,801	3,452	3,618
Yunnan	1,121	1,308	1,357	1,399	1,402	1,497	1,549	1,557	1,771	1,959	2,018	2,031	2,037	4,112	4,105	4,143	4,252
Tibet	0	0	0	0	0	0	0	0	0	0	0	0	0	93	86	95	98
Shaanxi	1,825	1,848	1,800	1,803	1,874	1,892	1,832	1,760	1,683	2,797	2,702	2,603	2,580	4,070	3,760	3,650	3,763
Gansu	1,276	1,190	1,198	1,115	1,077	1,082	1,058	1,027	1,062	1,162	1,297	1,350	1,443	2,473	2,290	2,341	2,532
Qinghai	243	214	210	170	169	202	239	244	252	342	520	559	566	597	611	603	691
Ningxia	224	229	212	228	270	270	301	332	363	411	529	619	650	938	884	819	842
Xinjiang	821	755	691	455	580	597	675	798	863	1,184	2,054	2,175	2,132	1,830	1,938	2,095	1,771
Unknown region	0	0	0	0	0	0	0	0	1,784	0	0	0	0	0	0	0	0
Our thermal power plants	640	848	910	1,048	1,055	1,122	1,205	1,402	138	74	45	37	31	1,828	1,820	1,853	1,908
CESD thermal power plants	n.a.	n.a.	n.a.	1,033	1,077	1,158	1,196	1,403	1,571	1,715	1,742	1,715	1,642	1,828	1,824	1,853	1,908
Our total	55,855	65,282	70,222	70,187	68,191	69,323	70,457	70,093	76,501	104,058	108,598	109,149	111,124	153,027	147,996	147,657	154,630
CESD total	74,097	70,978	70,944	71,377	70,797	69,665	70,462	70,514	76,185	106,457	110,373	110,905	112,799	153,027	147,996	147,657	154,633

Data for CESD are collected from China Statistical Yearbook on Environment and China Environment Yearbook. Total firm numbers are retrieved from tables grouped by sectors. Total number of CESD thermal power plants are collected from Annual Statistic Report on Environment in China.

Before aggregating the micro-level environmental data, deeper investigations of the whole dataset have been conducted in order to revise the abnormal data records and improve the data quality. There are typically two types of errors exist for the abnormal data record. The first type is missing data error and the other one is measurement error. The missing data error will lead to underestimation of pollutants’ values and the measurement error will lead to both underestimation and overestimation of pollutants’ values. We manually checked each firm’s time series data to revise these two issues. For the missing data, if a water-polluting firm has both COD and NH_3_-N discharge data in all year but lacks NH_3_-N discharge data in one specific year, then the missing NH_3_-N discharge data is identified and will be filled with the interpolation value. For the measurement error, it is likely that firms may report incorrect data by using different units. For example, if one firm reports the data in unit of gram instead of kilogram which is required by the regulations, then data value recorded in the database will be 1,000 times higher than the true value. This data will be identified as abnormal data and be revised to its reasonable level.

The raw two-dimensional environmental data can be calculated based on revised CIESD as follows:

1 $${\bar{X}}_{r,j}^{c}=\sum _{i}{x}_{r,j,i}^{c}$$

where ${x}_{r,j,i}^{c}$ is revised environmental data of firm *i* of sector *j* in region *r*. ${\bar{X}}_{r,j}^{c}$ represents raw macro-level environmental data of sector *j* in region *r*. Superscript *c* represents type of indicator, i.e. discharge or removal. We estimate the missing ${\bar{X}}_{r,j}^{c}$ by extrapolating the ratio of each sector among all regions, and the ratio is defined as follows:

2 $$Rati{o}_{r,j}^{c}=\frac{{\bar{X}}_{r,j}^{c}}{{\sum }_{r}{\bar{X}}_{r,j}^{c}}$$

Since micro-level environmental data in 2015 is not available, we use the structure ratios of 2014 as the prior structure information for 2015.

### Collection of macro-level environmental data

In this study, we collect macro-level environmental data from China Statistical Yearbook on Environment for year from 2001 to 2015 and China Environment Yearbook for year from 1998–2000. Environmental data in the yearbook are compiled from the CESD, which is collected and processed according to the environmental statistical system (ESS). The ESS was adjusted at the beginning of each Five-Year Plan period to improve the support the environmental statistical work and to improve the quality of environmental statistical data. Within the time period of our database, the ESS has been adjusted four times. These adjustments raise three biggest challenges for this study to construct the CIESD.

The first challenge is that not all pollutants are included in the CESD from 1998 to 2015. For example, NH_3_-N was reported since the Tenth Five-Year Plan (started from 2001) and NO_X_ was reported since the Eleventh Five-Year Plan (started from 2006). Due to the data availability, environmental data of these two pollutants are covered since the forementioned period. Table 2 reports whether each pollutant is reported and is listed as the major pollutant in four Five-Year Plan periods.

**Table 2:** Brief summary of pollutants during four Five-Year Plan periods.

	Ninth Five-Year Plan (1998–2000)		Tenth Five-Year Plan (2001–2005)		Eleventh Five-Year Plan (2006–2010)		Twelfth Five-Year Plan (2011–2014)	
	Reported	Major pollutant	Reported	Major pollutant	Reported	Major pollutant	Reported	Major pollutant
COD	Yes	Yes	Yes	Yes	Yes	Yes	Yes	Yes
SO_2_	Yes	Yes	Yes	Yes	Yes	Yes	Yes	Yes
NH_3_-N			Yes	Yes	Yes		Yes	Yes
NO_X_					Yes		Yes	Yes

Information of key pollutants are collected by authors from China’s Total Emission Control of Major Pollutants in each Five-Year Plan period.

The second challenge is that indicators of pollutants are not consistent within the whole data period. According to the ESS, industrial emission sources are divided into major sources and non-major sources. Only major sources are covered by the CESD, and they are expected to account for at least 85% of total annual discharges of major pollutants. When compiling the environmental statistical data from 1998 to 2010, regional removal data, sectoral discharge data and sectoral removal data are just the summation value of major sources. However, regional discharge data are adjusted to include non-major sources’ discharge data which are estimated by using certain estimation methods. As a result, the national data aggregated from regional data are typically larger than that aggregated from sectoral data. Besides, removal data are no longer reported according to the new ESS since the beginning of Twelfth Five-Year Plan. Instead, number of pollutants produced are reported in the CESD as well as in the yearbooks. Both the new production data and the discharge data are adjusted to include non-major sources. Table 3 reports the comparison results of regional aggregation to sectoral aggregation. For the period from 2011 to 2015, we calculate the pollutants’ removal data by subtracting discharge data from production data. Inconsistent treatments of non-major sources in regional and sectoral data will be balanced using cross-entropy method in this study.

**Table 3:** Ratios of regional aggregation to sectoral aggregation for three indicators of four pollutants, 1998–2015.

	Data type	1998	1999	2000	2001	2002	2003	2004	2005	2006	2007	2008	2009	2010	2011	2012	2013	2014	2015
COD	Discharge	1.00	1.01	1.01	1.15	1.13	1.16	1.13	1.12	1.17	1.13	1.13	1.16	1.19	1.10	1.11	1.12	1.13	1.15
	Removal	1.00	1.00	1.00	1.00	1.00	1.00	1.00	1.00	1.00	1.00	1.00	1.00	1.00					
	Production														1.01	1.06	1.06	1.06	1.07
SO_2_	Discharge	1.00	1.02	1.01	1.11	1.11	1.20	1.08	1.10	1.09	1.09	1.08	1.10	1.09	1.07	1.07	1.09	1.10	1.11
	Removal	1.00	1.00	1.00	1.00	1.00	1.01	1.00	1.00	0.99	1.00	1.00	1.00	1.00					
	Production														1.02	1.04	1.04	1.05	1.05
NH_3_-N	Discharge				1.14	1.11	1.09	1.09	1.09	1.13	1.11	1.11	1.12	1.11	1.07	1.09	1.09	1.10	1.11
	Removal				1.00	1.00	1.00	1.00	1.00	1.00	1.00	1.00	1.00	1.00					
	Production														1.01	1.06	1.05	1.05	1.07
NO_X_	Discharge									1.09	1.09	1.09	1.07	1.07	1.04	1.05	1.06	1.06	1.08
	Removal									1.00	1.00	1.00	1.00	1.00					
	Production														1.04	1.05	1.05	1.06	1.07

Raw data are collected from China Statistical Yearbook on Environment and China Environment Yearbook. Ratios are calculated by authors.

**Fig. 1:**
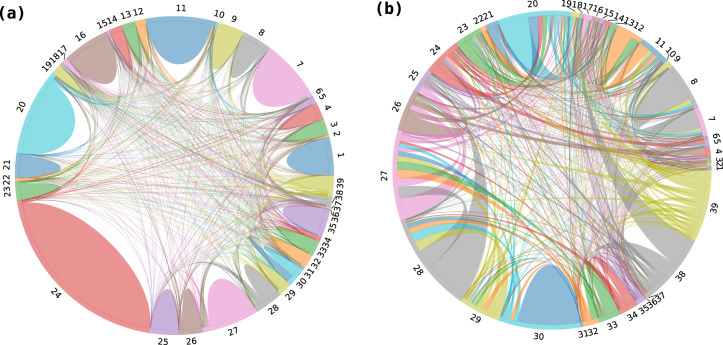
Revisions of firms’ 2-digit sector codes. (**a**) Whole dataset; (b) Only firms whose 2-digit sector codes are revised. Each node in the chord diagram represents one 2-digit sector and arcs represent directions and numbers of firms that have been revised from one sector to another.

The third challenge is that the classifications of sectors reported in official statistics have been changed three times within the data period. Classifications of sectors are based on Classification Standards of National Economic Industries (CSNEI) which includes 2-digit, 3-digit and 4-digit codes for all sectors and sub-sectors. Three versions of CSNEI used for classification are GB/T 4754-1994, GB/T 4754-2002 and GB/4754-2011. In the period from 1998 to 2000, all firms are classified into 20 sectors (including cement manufacturing as a sub-sector). The classification changed for the first time in 2001 and 2002, all firms are classified into 43 sectors (including cement manufacturing and thermal power as sub-sectors). Then the classification changed for the second time from 2003 to 2010, all firms are classified into 42 sectors (including cement manufacturing and thermal power as sub-sectors). Finally, the classification changed for the third time from 2011 to 2015, all firms are classified into 42 sectors (including four separate sub-sectors with regional data). To make the data comparable across different years, we coordinate all sectors into 39 sectors labelled from 1 to 39. Table 4 reports the concordance of industrial sectors and the first column is the coordinated sector number. We also find that for some firms, they have different 2-digit sector codes recorded in CIESD and Annual Survey of Industrial Enterprises Database (ASIED). Since 2-digit sector codes in ASIED are more consistent to the sector classifications of official statistics released by the National Bureau of Statistics of China. We match the firms in CIESD and ASIED, and revise firms’ 2-digit sector codes according to the ASIED before the concordance of sectors. Besides, firms classified as “Others” in CIESD are all checked and revised manually. The chord diagram in Fig. 1(a) illustrates the changes of all firms’ sector codes in CIESD. We can see that most of firms’ sector codes in the CIESD are not revised or remain the same 2-digit sector codes after revision. If we take a close look at firms whose 2-digit sector codes are revised, interchanges occur in most sectors except for the “Others” sector whose 2-digit sector code is 39. Since all firms originally classified in the “Others” sector have been manually checked and revised, they are now assigned the correct 2-digit sector codes.

**Table 4:** Concordance of industrial sectors.

No	Concordant sector	1998–2000		2001–2002		2003–2010		2011–2014	
	Sectors	Sectors	2-digit code (1994)	Sectors	2-digit code (1994)	Sectors	2-digit code (2002)	Sectors	2-digit code (2011)
1	Mining and Washing of Coal	Mining and quarrying (part)	06	Coal Mining and Dressing	06	Mining and Washing of Coal	06	Mining and Washing of Coal	06
2	Extraction of Petroleum and Natural Gas		07	Petroleum and Natural Gas Extraction	07	Extraction of Petroleum and Natural Gas	07	Extraction of Petroleum and Natural Gas	07
3	Mining and Processing of Ferrous Metal Ores		08	Ferrous Metals Mining and Dressing	08	Mining and Processing of Ferrous Metal Ores	08	Mining and Processing of Ferrous Metal Ores	08
4	Mining and Processing of Non-ferrous Metal Ores		09	Nonferrous Metals Mining and Dressing	09	Mining and Processing of Non-ferrous Metal Ores	09	Mining and Processing of Non-ferrous Metal Ores	09
5	Mining and Processing of Non-metal Ores		10	Nonmetal Minerals Mining and Dressing	10	Mining and Processing of Non-metal Ores	10	Mining and Processing of Non-metal Ores	10
6	Mining of Other Ores		11	Other Minerals Mining and Dressing	11	Mining of Other Ores	11	Ancillary Activities for Exploitation	11
								Mining of Other Ores	12
7	Processing of Food from Agricultural Products	Food, beverage, and tobacco processing	13	Food Processing	13	Processing of Food from Agricultural Products	13	Processing of Food from Agricultural Products	13
8	Manufacture of Foods		14	Food Manufacturing	14	Manufacture of Foods	14	Manufacture of Foods	14
9	Manufacture of Wine, Drinks and Refined Tea		15	Beverage Manufacturing	15	Manufacture of Beverages	15	Manufacture of Wine, Drinks and Refined Tea	15
10	Manufacture of Tobacco		16	Tobacco Processing	16	Manufacture of Tobacco	16	Manufacture of Tobacco	16
11	Manufacture of Textile	Textile Industry	17	Textile Industry	17	Manufacture of Textile	17	Manufacture of Textile	17
12	Manufacture of Textile Wearing and Apparel	Other Industries (part)	18	Garments and Other Fiber Products	18	Manufacture of Textile Wearing Apparel, Footwear, and Caps	18	Manufacture of Textile Wearing and Apparel	18
13	Manufacture of Leather, Fur, Feather and Related Products and Footwear	Leather, furs, down and related products	19	Leather, Furs, Down and Related Products	19	Manufacture of Leather, Fur, Feather and Related Products	19	Manufacture of Leather, Fur, Feather and Related Products and Footwear	19
14	Processing of Timber, Manufacture of Wood, Bamboo,Rattan, Palm, and Straw Products	Other Industries (part)	20	Timber Processing, Bamboo, Cane, Palm Fiber and Straw Products	20	Processing of Timber, Manufacture of Wood, Bamboo, Rattan, Palm, and Straw Products	20	Processing of Timber, Manufacture of Wood, Bamboo, Rattan, Palm, and Straw Products	20
15	Manufacture of Furniture		21	Furniture Manufacturing	21	Manufacture of Furniture	21	Manufacture of Furniture	21
16	Manufacture of Paper and Paper Products	Papermaking and Paper Products	22	Papermaking and Paper Products	22	Manufacture of Paper and Paper Products	22	Manufacture of Paper and Paper Products	22
17	Printing,Reproduction of Recording Media	Printing	23	Printing	23	Printing, Reproduction of Recording Media	23	Printing, Reproduction of Recording Media	23
18	Manufacture of Articles for Culture, Education and Sport Activity	Other Industries (part)	24	Cultural, Educational and Sports Goods	24	Manufacture of Articles for Culture, Education and Sport Activity	24	Manufacture of Articles for Culture, Education and Sport Activity	24
19	Processing of Petroleum, Coking, Processing of Nuclear Fuel	Petroleum Processing and Coking	25	Petroleum Processing and Coking	25	Processing of Petroleum, Coking, Processing of Nuclear Fuel	25	Processing of Petroleum, Coking, Processing of Nuclear Fuel	25
20	Manufacture of Raw Chemical Materials and Chemical Products	Raw Chemical Materials and Chemical Products	26	Raw Chemical Materials and Chemical Products	26	Manufacture of Raw Chemical Materials and Chemical Products	26	Manufacture of Raw Chemical Materials and Chemical Products	26
21	Manufacture of Medicines	Medical and Pharmaceutical Products	27	Medical and Pharmaceutical Products	27	Manufacture of Medicines	27	Manufacture of Medicines	27
22	Manufacture of Chemical Fibers	Chemical Fiber	28	Chemical Fiber	28	Manufacture of Chemical Fibers	28	Manufacture of Chemical Fibers	28
23	Manufacture of Rubber and Plastic	Rubber Products	29	Rubber Products	29	Manufacture of Rubber	29	Manufacture of Rubber and Plastic	29
		Plastic Products	30	Plastic Products	30	Manufacture of Plastic	30		
24	Manufacture of Non-metallic Mineral Products	Nonmetal Mineral Products	31	Nonmetal Mineral Products	31	Manufacture of Non-metallic Mineral Products	31	Manufacture of Non-metallic Mineral Products	30
25	Smelting and Pressing of Ferrous Metals	Smelting and Pressing Of Ferrous Metals	32	Smelting and Pressing Of Ferrous Metals	32	Smelting and Pressing of Ferrous Metals	32	Smelting and Pressing of Ferrous Metals	31
26	Smelting and Pressing of Non-ferrous Metals	Smelting and Pressing Of Non-ferrous Metals	33	Smelting and Pressing Of Non-ferrous Metals	33	Smelting and Pressing of Non-ferrous Metals	33	Smelting and Pressing of Non-ferrous Metals	32
27	Manufacture of Metal Products	Metal Products	34	Metal Products	34	Manufacture of Metal Products	34	Manufacture of Metal Products	33
		Machine, Electric, Machinery and Electronic Equipment Manufacturing	39	Weapons and Ammunition Manufacturing	39				
28	Manufacture of General Purpose Machinery		35	Ordinary Machinery Manufacturing	35	Manufacture of General Purpose Machinery	35	Manufacture of General Purpose Machinery	34
29	Manufacture of Special Purpose Machinery		36	Special Purpose Equipment Manufacturing	36	Manufacture of Special Purpose Machinery	36	Manufacture of Special Purpose Machinery	35
30	Manufacture of Automobile		37	Transport Equipment Manufacturing	37	Manufacture of Transport Equipment	37	Manufacture of Automobile	36
31	Manufacture of Railway, Shipbuilding, Aerospace and Other Transportation Equipment							Manufacture of Railway, Shipbuilding, Aerospace and Other Transportation Equipment	37
32	Manufacture of Electrical Machinery and Equipment		40	Electric Equipment and Machinery	40	Manufacture of Electrical Machinery and Equipment	39	Manufacture of Electrical Machinery and Equipment	38
33	Manufacture of Computers, Communication, and Other Electronic Equipment		41	Electronic and Telecommunications Equipments	41	Manufacture of Communication Equipment, Computers and Other Electronic Equipment	40	Manufacture of Computers, Communication, and Other Electronic Equipment	39
34	Manufacture of Measuring Instrument		42	Instrument, Meters, Cultural and Office Machinery Manufacturing	42	Manufacture of Measuring Instruments and Machinery for Cultural Activity and Office Work	41	Manufacture of Measuring Instrument	40
35	Production and Supply of Electric Power and Heat Power	Production and Supply of Electric Power, Gas and Water	44	Electric Power, Steam and Hot Water Productions	44	Production and Distribution of Electric Power and Heat Power	44	Production and Supply of Electric Power and Heat Power	44
36	Production and Supply of Gas		45	Gas Production and Supply	45	Production and Distribution of Gas	45	Production and Supply of Gas	45
37	Production and Supply of Water		46	Tap Water Production and Supply	46	Production and Distribution of Water	46	Production and Supply of Water	46
38	Other Manufactures	Other Industries (part)	43	Other Manufactures	43	Manufacture of Artwork and Other Manufacturing	42	Other Manufactures	41
						Recycling and Disposal of Waste	43	Utilization of Waste Resources	42
								Metal Products, Machinery and Equipment Repair	43
39	Others	Mining and quarrying (part)	12	Logging and Transport of Timber and Bamboo	12	Others	99	Others	99
		Other Industries (part)	99	Others	99				

The mapping rules are set by authors after comparing sector names and descriptions manually. Complete list of 4-digit sector code mapping rules used for concordance are provided in the Supplementary Table 1.

Moreover, since China has conducted the first National Census of Pollution Sources (NCPS) in 2007, the ESS has significantly changed according to the census results. As a result, there are two special concerns we must pay attention to when using the environmental data. Firstly, the ESS relied heavily on firms’ self-reported data to determine the major emission sources before 2011. After adopting the complete emission source list provided by the NCPS, total number of firms included in the CESD increased significantly in 2011 and keeps stable thereafter. As a result, it is not surprising that total discharge data jump at 2011 as well. Secondly, a large number of discharge coefficients have been updated by the NCPS and they have been adopted in the ESS adjusted in 2011. Consequently, some sectors pollution levels also changed greatly. If researchers conduct empirical studies by using panel models, these systematic changes can be captured by the sector and time fixed effects. While if researchers use methods such as index decomposition, data envelope analysis and so on, the forementioned concerns should be treated carefully.

### Balancing the environmental data

We use a two-step adjustment procedure to adjust the raw two-dimensional environmental data to construct the final CIESD. In the first step, the raw value is adjusted by using the following formula:

3 $${\widetilde{X}}_{r,j}^{c}={\bar{X}}_{r,j}^{c}\times \frac{{Y}_{r}^{c}}{{\sum }_{j}{\bar{X}}_{r,j}^{c}}$$

where ${\widetilde{X}}_{r,j}^{c}$ represents the adjusted raw value and ${Y}_{r}^{c}$ represents aggregated macro-level environmental data for region *r*. The second term in the right-hand side of formula (3) is the region-specific average scale ratio to add non-major emission sources. Sectors in the same region are assumed to be scaled up proportionally. This step adjusts the aggregated regional raw data and make them consistent to the macro-level environmental data.

In the second step, we use the cross-entropy method to balance the two-dimensional data matrix^31^. In this paper, we assume the regional distributions of the environmental data within each sector are more reliable. Thus, we define the prior coefficients ${\widetilde{a}}_{r,j}^{c}$ and the new coefficients to be estimated ${\widehat{a}}_{r,j}^{c}$ as follows:

4 $${\widetilde{a}}_{r,j}^{c}=\frac{{\widetilde{X}}_{r,j}^{c}}{{\sum }_{r}{\widetilde{X}}_{r,j}^{c}}$$

5 $${\widehat{a}}_{r,j}^{c}=\frac{{\widehat{X}}_{r,j}^{c}}{{\sum }_{r}{\widetilde{X}}_{r,j}^{c}}$$

where ${\widehat{X}}_{r,j}^{c}$ is the new environmental data to be estimated. Then the objective function is defined as follows:

6 $$\mathop{\min }\limits_{\left\{{\widehat{a}}_{r,j}^{c}\right\}}\sum _{r}\sum _{j}{\widehat{a}}_{r,j}^{c}ln\frac{{\widehat{a}}_{r,j}^{c}}{{\widetilde{a}}_{r,j}^{c}}$$

subject to:

7 $$\sum _{r}\sum _{j}\left({\widehat{a}}_{r,j}^{c}\sum _{r}{\widetilde{X}}_{r,j}^{c}\right)=\sum _{r}{Y}_{r}^{c}$$

8 $$\sum _{r}{\widehat{a}}_{r,j}^{c}=1\;{\rm{and}}\;0\le {\widehat{a}}_{r,j}^{c}\le 1$$

The solution for ${\widehat{a}}_{r,j}^{c}$ is obtained by solving the optimization problem from formula (6)-(8) and the final estimated environmental data are obtained as follows:

9 $${\widehat{X}}_{r,j}^{c}={\widehat{a}}_{r,j}^{c}\sum _{r}{\widetilde{X}}_{r,j}^{c}$$

## Data Records

Our data records are available through Figshare in format of Excel file from the repository: 10.6084/m9.figshare.16846966^32^. Table 5 presents the structure of the environmental data for each year by region and by sector. Each matrix includes 31 regions and 39 sectors. Totally 122 matrices are included in the database. Of these,

- 72 matrices are discharge and removal data for COD and SO_2_ from 1998 to 2015;
- 30 matrices are discharge and removal data for NH_3_-N from 2001 to 2015;
- 20 matrices are discharge and removal data for NO_X_ from 2006 to 2015.

**Table 5:** The structure of the environmental data by region and by sector.

	Year	Sector 1	Sector 2	…	Sector 39	Total
Region 1						
Region 2						
…						
Region 31						
Total						

The names of region 1 to 31, sector 1 to 39 can be found in Tables 1 and 4, respectively.

Unit for all environmental data is kilogram (kg).

## Technical Validation

### Creditability of firm-level data

The prior information to construct the environmental data matrix are aggregated from the firm-level data, thus it is important to validate the creditability of these micro-level data. Since the yearbooks are the only authoritative data sources and sectoral data in the yearbooks only cover major emission sources, we compare data in our database to yearbooks’ data. In the Supplementary Fig. 1, we provided the comparison information for all pollutants by sector and by year. For years from 2000 to 2014, aggregation value for each sector in our database are very close to that in the yearbook. For years 1998 and 1999, although total observations are less than the yearbook, ratios that aggregation value in our database divided by total value in the yearbook are similar across sectors. Therefore, firm-level data in our database is credible and it is appropriate to calculate the coefficients in formula (4) by sector.

### Validation of balanced results

**Fig. 2:**
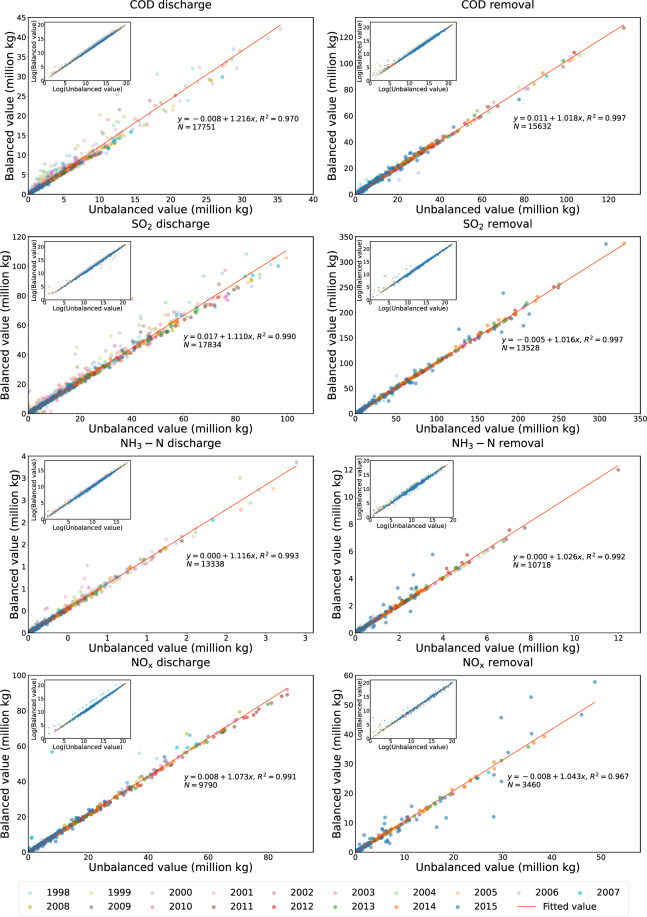
Comparison of unbalanced value and balanced value from 1998 to 2015. (**a**) COD discharge; (**b**) COD removal; (**c**) SO_2_ discharge; (**d**) SO_2_ removal; (**e)** NH_3_-N discharge; (**f**) NH_3_-N removal; (**g**) NO_X_ discharge; (**h**) NO_X_ removal. Fitted values are obtained from pooled OLS regressions.

Our estimation procedure aims to adjust the environmental data and make them consistent to the regional aggregations. Since regional aggregations include non-major emission sources, estimated environmental data have been systematically scaled up. For each year, we run the following two-way fixed effect regression to investigate adjustments from the original data to the estimated data:

10 $${\widehat{X}}_{r,j}^{c}={\alpha }_{r}+{\gamma }_{j}+{\beta }^{c}{\bar{X}}_{r,j}^{c}+{\varepsilon }_{r,j}^{c}$$

where *β*^*c*^ is the slope coefficient and ${\varepsilon }_{r,j}^{c}$ is the noise term which follows normal distribution, ${\varepsilon }_{r,j}^{c} \sim N\left(0,{\sigma }_{c}^{2}\right)$. Table 6 presents estimated slope coefficients and standard deviations for different pollutants. All coefficients are statistically significant at 1% level. Table 7 presents adjusted *R*^2^ for different pollutants and most of them are greater than 0.99. Figure 2 provides visual illustrations of the comparisons of unbalanced value and balanced value for four types of pollutants.

**Table 6:** Estimated slope coefficients for different pollutants by year.

	Data type	1998	1999	2000	2001	2002	2003	2004	2005	2006	2007	2008	2009	2010	2011	2012	2013	2014	2015
COD	Discharge	1.5060 (0.1498)	1.2950 (0.0468)	1.2685 (0.0534)	1.1194 (0.0122)	1.1084 (0.0138)	1.1311 (0.0201)	1.1172 (0.0212)	1.1161 (0.0176)	1.1307 (0.0236)	1.0989 (0.0162)	1.0991 (0.0167)	1.1202 (0.0217)	1.1472 (0.0292)	1.0876 (0.0113)	1.0952 (0.0140)	1.0984 (0.0159)	1.1087 (0.0180)	1.1123 (0.0219)
	Removal	1.2875 (0.1801)	1.0478 (0.0148)	1.0137 (0.0035)	1.0043 (0.0017)	1.0010 (0.0022)	1.0016 (0.0014)	1.0004 (0.0002)	1.0010 (0.0002)	0.9987 (0.0024)	0.9974 (0.0006)	0.9983 (0.0004)	0.9971 (0.0006)	0.9967 (0.0007)	1.0000 (0.0000)	1.0371 (0.0097)	1.0372 (0.0091)	1.0378 (0.0094)	1.0509 (0.0160)
SO_2_	Discharge	1.4534 (0.0705)	1.2692 (0.0267)	1.2395 (0.0211)	1.0985 (0.0067)	1.1045 (0.0056)	1.1929 (0.0123)	1.0889 (0.0044)	1.0712 (0.0031)	1.0943 (0.0039)	1.0663 (0.0024)	1.0692 (0.0027)	1.0855 (0.0038)	1.0746 (0.0037)	1.0590 (0.0039)	1.0657 (0.0045)	1.0777 (0.0058)	1.0900 (0.0075)	1.0982 (0.0088)
	Removal	1.2443 (0.0846)	1.1366 (0.0330)	1.0100 (0.0012)	0.9209 (0.0111)	0.9882 (0.0020)	0.9079 (0.0117)	0.9143 (0.0108)	0.9607 (0.0057)	1.0162 (0.0009)	0.9979 (0.0004)	0.9982 (0.0005)	0.9982 (0.0004)	0.9972 (0.0005)	1.0000 (0.0000)	1.0183 (0.0007)	1.0320 (0.0017)	1.0356 (0.0023)	1.0211 (0.0015)
NH_3_-N	Discharge				1.1207 (0.0137)	1.1130 (0.0126)	1.0787 (0.0083)	1.0841 (0.0111)	1.1057 (0.0121)	1.1213 (0.0169)	1.0861 (0.0108)	1.0792 (0.0102)	1.0847 (0.0116)	1.1048 (0.0149)	1.0642 (0.0075)	1.0815 (0.0098)	1.0842 (0.0091)	1.0874 (0.0098)	1.0903 (0.0112)
	Removal				0.9996 (0.0022)	1.0025 (0.0004)	1.0022 (0.0003)	0.9999 (0.0001)	1.0024 (0.0005)	1.0221 (0.0048)	1.0056 (0.0018)	1.0069 (0.0019)	0.9994 (0.0001)	0.9995 (0.0001)	1.0000 (0.0000)	1.0654 (0.0056)	1.0452 (0.0035)	1.0423 (0.0036)	1.1348 (0.0134)
NO_X_	Discharge									1.0837 (0.0042)	1.0371 (0.0037)	1.0742 (0.0034)	1.0631 (0.0028)	1.0572 (0.0021)	1.0405 (0.0014)	1.0441 (0.0016)	1.0529 (0.0021)	1.0637 (0.0031)	1.0803 (0.0046)
	Removal									1.0116 (0.0020)	0.9989 (0.0005)	0.9992 (0.0002)	0.9794 (0.0102)	1.0000 (0.0000)	0.9964 (0.0003)	1.0446 (0.0040)	1.0270 (0.0010)	1.0304 (0.0007)	1.0481 (0.0017)

Standard deviations are provided in the parenthesis.

**Table 7:** Adjusted overall R2 for different pollutants by year.

	Data type	1998	1999	2000	2001	2002	2003	2004	2005	2006	2007	2008	2009	2010	2011	2012	2013	2014	2015
COD	Discharge	0.9531	0.9883	0.9879	0.9970	0.9962	0.9960	0.9959	0.9976	0.9954	0.9983	0.9984	0.9973	0.9944	0.9991	0.9988	0.9986	0.9982	0.9973
	Removal	0.9460	0.9986	0.9999	0.9990	0.9991	0.9988	0.9992	1.0000	0.9955	0.9999	1.0000	1.0000	0.9999	1.0000	0.9996	0.9996	0.9996	0.9982
SO_2_	Discharge	0.9852	0.9947	0.9959	0.9992	0.9990	0.9972	0.9993	0.9997	0.9986	0.9997	0.9998	0.9996	0.9995	0.9998	0.9997	0.9996	0.9994	0.9991
	Removal	0.9778	0.9938	0.9999	0.9983	0.9998	0.9974	0.9975	0.9991	0.9933	0.9999	1.0000	1.0000	1.0000	1.0000	1.0000	0.9992	0.9986	0.9991
NH_3_-N	Discharge				0.9976	0.9983	0.9991	0.9980	0.9976	0.9966	0.9986	0.9989	0.9987	0.9974	0.9996	0.9993	0.9992	0.9991	0.9984
	Removal				0.9993	1.0000	1.0000	1.0000	1.0000	0.9966	0.9995	0.9996	1.0000	1.0000	1.0000	0.9994	0.9998	0.9998	0.9922
NO_X_	Discharge									0.9988	0.9961	0.9996	0.9998	0.9998	0.9999	0.9999	0.9999	0.9998	0.9994
	Removal									0.9993	1.0000	1.0000	0.9981	1.0000	1.0000	0.9997	0.9999	0.9999	0.9965

### Comparison with provincial official statistics

**Fig. 3:**
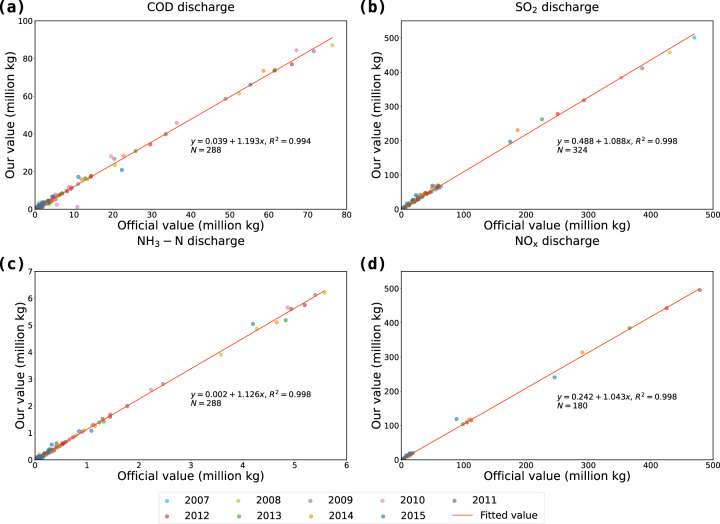
Comparison of Zhejiang’s official value and our value. (**a**) COD discharge; (**b**) SO_2_ discharge; (**c**) NH_3_-N discharge; (**d**) NO_X_ discharge. Fitted values are obtained from pooled OLS regressions.

Among all provinces in China, only Zhejiang province reports sectoral level environmental data. We collect sectoral level data for four types of pollutants from *Zhejiang Statistical Yearbook on Environment* and *Zhejiang Natural Resources and Statistical Yearbook on Environment* and coordinate all industrial sectors according to Table 4. Figure 3 provides visual illustrations of the comparisons of official value and our value for four types of pollutants. We can see that observations for all pollutants are well fitted and OLS results in Fig. 3 are highly consistent to those provided in Fig. 2.

### Comparison with national aggregation data

**Fig. 4:**
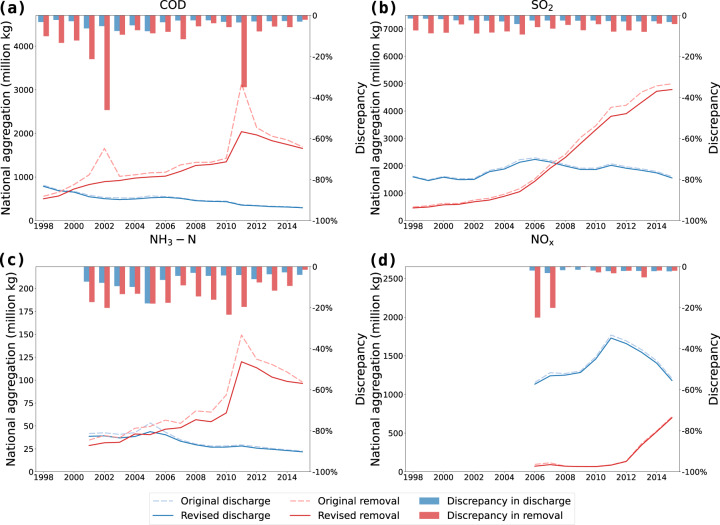
Comparison of original and revised national aggregation data from 1998 to 2015. (**a**) COD; (**b**) SO_2_; (**c**) NH_3_-N; (**d**) NO_X_. Discrepancies are shown in bars on the secondary axis.

The national aggregation data of four types of pollutants have been revised due to the correction of micro-level firm data. Figure 4 presents the comparisons of original and revised national aggregation data. Results show that the revised national aggregation data are smoother than the original data over the time. Abnormal data such as national COD removal data in 2002 and 2012 have been corrected. Almost all discharge and removal data of four types of pollutants have been adjusted downward for all years. Removal data have higher reduction rates than discharge data.

## Supplementary information

Supplementary Figure 1

Supplementary Table 1

## Data Availability

In this study, we use the General Algebraic Modeling System (GAMS) to conduct the cross-entropy estimation, and MINOS solver is used to conduct the nonlinear optimization tasks. All codes used for analysis are available in the public GitHub repository: https://github.com/qianhaoqi/China-Industrial-Environmental-Database.
