# Supplementary material for: China industrial environmental database 1998–2015
Source: Sci Data. 2022 Jun 1;9:259. doi: 10.1038/s41597-022-01362-x (PMC9160261; doi:10.1038/s41597-022-01362-x)
Supplement: Supplementary file 1 — Supplementary Figure 1 [file 41597_2022_1362_MOESM1_ESM.docx]

Supplementary Information for

**China Industrial Environmental Database 1998-2015**

**Authors**

Haoqi Qian^1,2,10,*^, Feizhou Ren^3,4,10^, Yanran Gong^5^, Rong Ma^4^, Wendong Wei^6,7^, Libo Wu^4,8,9,*^

**Affiliations**

1. Institute for Global Public Policy and MOE Laboratory for National Development and Intelligent Governance, Fudan University, Shanghai, 200433, China

2. LSE-Fudan Research Centre for Global Public Policy, Fudan University, Shanghai, 200433, China

3. Shanghai Pudong Development Bank, Shanghai, 200002, China

4. School of Economics, Fudan University, Shanghai, 200433, China

5. School of Data Science, Fudan University, Shanghai, 200433, China

6. School of International and Public Affairs, Shanghai Jiao Tong University, Shanghai, 200030, China

7. SJTU-UNIDO Joint Institute of Inclusive and Sustainable Industrial Development, Shanghai Jiao Tong University, Shanghai, 200030, China;

8. Institute for Big Data, Fudan University, Shanghai, 200433, China

9. Shanghai Institute for Energy and Carbon Neutrality Strategy, Fudan University, Fudan University, Shanghai, 200433, China

10. These authors contributed equally

*corresponding author(s): Haoqi Qian ([qianhaoqi@fudan.edu.cn](mailto:wendongwei@sjtu.edu.cn)), Libo Wu ([wulibo@fudan.edu.cn](mailto:wulibo@fudan.edu.cn))

**This file includes:**

Supplementary Figure 1


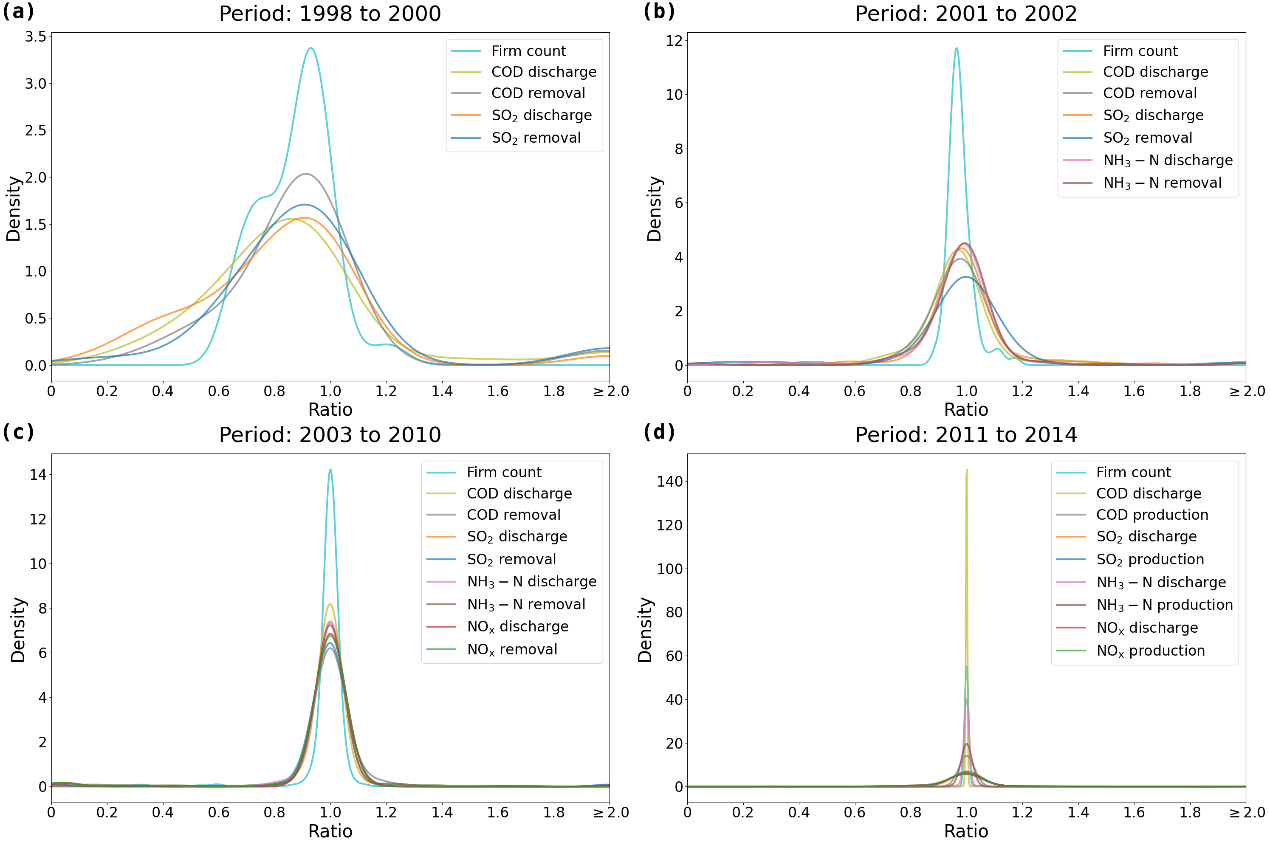


Figure 1. **Distributions of ratios of aggregated CIESD data to yearbook data.** (a) Period 1998-2000; (b) Period 2001-2002; (c) Period 2003-2010; (d) Period 2011-2014. Distributions are estimated by using Gaussian Kernel Density Estimation.
